# Effect of microplastic on sorption, toxicity, and mineralization of 2,4-dichlorophenoxyacetic acid ionic liquids

**DOI:** 10.1007/s00253-024-13353-6

**Published:** 2024-11-26

**Authors:** Natalia Lisiecka, Marta Woźniak-Karczewska, Anna Parus, Maria Simpson, Robert Frankowski, Agnieszka Zgoła-Grześkowiak, Katarzyna Siwińska-Ciesielczyk, Michał Niemczak, Christian Eberlein, Hermann J. Heipieper, Łukasz Chrzanowski

**Affiliations:** 1 Institute of Chemical Technology and Engineering, Poznan University of Technology, Berdychowo 4, 60-965 Poznan, Poland; 2 Department of Molecular Environmental Biotechnology, Helmholtz Centre for Environmental Research – UFZ, Permoserstraße 15, 04318 Leipzig, Germany; 3 Institute of Chemistry and Technical Electrochemistry, Poznan University of Technology, Berdychowo 4, 60-965 Poznan, Poland

**Keywords:** Polyethylene, Herbicides, 2,4-D, *Pseudomonas putida* KT2440, Antimicrobial activity

## Abstract

**Abstract:**

Recently, there has been significant focus on microplastics in the environment, especially regarding their role in sorption–desorption processes of emerging contaminants, impacting pollutant migration between aquatic and terrestrial ecosystems. Notably, the newest pollutants in such environments are the herbicide formulations known as ionic liquids (ILs), which integrate the structure of classic herbicidal anion with surface-active cations acting as an adjuvant. In our study, we synthesized herbicidal ILs by combining 2,4-D anion with cetyltrimethylammonium [CTA] and didecyldimethylammonium [DDA] cations. We investigated whether ILs and the mixture of salts, when exposed to polyethylene (PE) microplastics, differ in properties. We analyzed their sorption on defined PE particles, evaluated toxicity on *Pseudomonas putida* KT2440 using trans/cis ratio of unsaturated fatty acids, and assessed biodegradability with OECD 301F standard test. Results indicate IL cations and anions behave as distinct entities, questioning IL synthesis feasibility. Hydrophobic adjuvants were found to adsorb onto PE microplastic surfaces (5–60% [CTA] > [DDA]), posing potential threats of surface-active xenobiotic accumulation. This highlights the need to explore microplastics’ role as sorbents of hazardous adjuvants in agriculture, potentially competing with humic acids and affecting xenobiotic bioavailability. Consequently, xenobiotics may persist longer in the environment, facilitated by microplastic mobility between aquatic and terrestrial ecosystems.

**Key points:**

*• Microplastics act as sorbents, accumulating xenobiotics and limiting biodegradation.*

*• Sorption of surfactant cations on microplastics reduces soil bacteria toxicity.*

*• Research confirms independent action of ions from ionic liquids in the environment.*

**Graphical abstract:**

**Figure:**
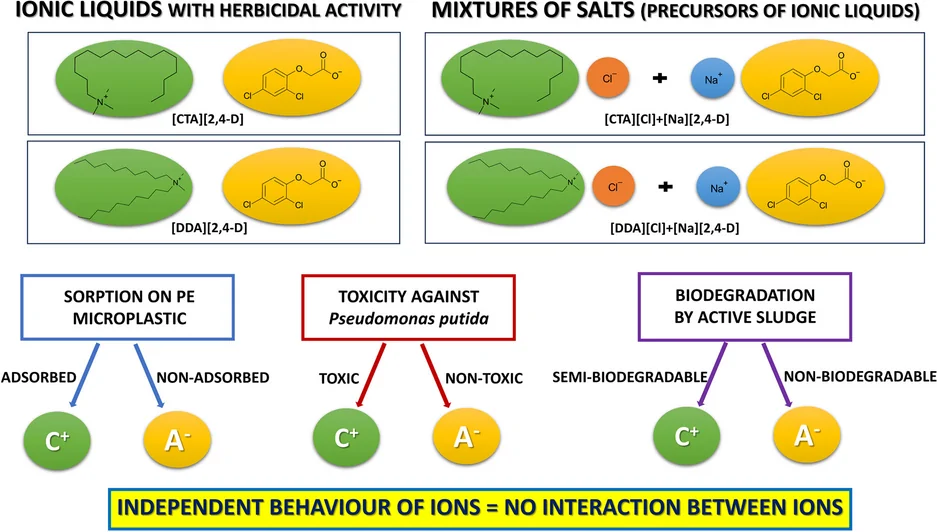

**Supplementary Information:**

The online version contains supplementary material available at 10.1007/s00253-024-13353-6.

## Introduction

In recent years, several scientific breakthroughs have provided uncertainties about whether ionic liquids (ILs) can be used as potential, greener alternatives instead of classically applied solvents (Pham et al. 2010; Bystrzanowska et al. 2019; Wilms et al. 2020b). Various toxicity studies suggest that many ILs can exhibit substantial toxicity toward cell lines (Hwang et al. 2018; Kumari et al. 2020; Musiał et al. 2021) and organisms at various tropic levels such as axenic cultures of microorganisms, microbial communities (Costa et al. 2014; Montalbán et al. 2016; Borkowski et al. 2018), fish (Dong et al. 2013; Du et al. 2014), or laboratory animals (Luo et al. 2009; Torrecilla et al. 2009; Yan et al. 2019). The aforementioned studies present a serious argument which postulates that it is impossible to explicitly describe ILs as potential, green alternatives for modern industry.

Toxicity studies serve as crucial indicators for predicting the future toxicity of the new cationic and anionic pair. Given the expectation that the herbicide in the form of ILs would decrease the overall toxicity, evaluations were conducted using various ILs (Gonçalves et al. 2007; Pham et al. 2010). During these toxicity studies, the integrity of the ionic pair was ignored; therefore, this aspect has recently been preliminarily tested and described by Lisiecka et al. (2023b, 2023a), Parus et al. (2022), and Woźniak-Karczewska et al. (2022). From an environmental point of view, it is therefore critical to consider whether the toxicity of the ionic pair is really analysed by solvating the IL in water. Or alternatively, should one perhaps assess the separate effects of each ion in the ionic solution? Since it is impossible to compare the toxicity of water-soluble and non-dissolvable salts (Appetecchi et al. 2006; Nacham et al. 2017), their toxicity should be analysed separately based on their varying properties.

Moreover, when performing toxicity studies, it should be noted that there is a direct correlation between hydrophobicity and toxicity of the tested compounds (Merianos 1991; Moussa et al. 1996; Russell et al. 2000; Heipieper et al. 2007). In the case of quaternary ammonium salts (QASs), combined with simple inorganic anions, the substance toxicity seems to result from the presence of the surface-active cation, whereas the anion does not have an effect on the toxicity (Piotrowska et al. 2017). Furthermore, the use of a specific anion can assist in a direct analysis of the effects of a single cation (Parus et al. 2022; Lisiecka et al. 2023b).

Herbicidal ILs often yield better results when composed of an herbicidal anion and a surface-active cation, rather than using adjuvants, which are often recognized as toxic additives in herbicidal formulations (Niemczak et al. 2020; Stachowiak et al. 2022). Notably, the conducted analysis did not consider the toxicity of specific ions, since the integrity of the ionic pair was not studied before.

Another aspect, beyond the toxicity, that one should make note of, is the possibility of an interaction between specific ions of the IL and the presence of a sorbent (Studzińska et al. 2008; Matzke et al. 2009; Mrozik et al. 2012). In the case of aqueous systems, it is particularly important to notice the occurrence of microplastics, which are identified as one of the main pollutions in the environment according to the European Environment Agency (European Environment Agency 2020) and are monitored by various scientists (Bandow et al. 2017; Wang et al. 2019; Akanyange et al. 2022; Yan et al. 2022).

In the case of agricultural soils, the introduction of synthetic polymers occurs through the application of plastic mulch, irrigation foils, and tapes, as well as sewage sludge utilized as fertilizers (Nizzetto et al. 2016; Weithmann et al. 2018; He et al. 2018; Wanner 2021). The most abundant plastics in agricultural soils are polyethylene, polyvinyl chloride, and ethyl acrylate (Sanchez-Hernandez 2019), which are subjected to degradation as a result of microparticle aging (Arthur et al. 2009; Jahnke et al. 2017; Alimi et al. 2018). Notably, the degradation rate of microplastics as well as herbicides and ILs varies significantly. Hence, the presence of their residues in the soil creates a realistic threat of an unwanted accumulation and interaction in the soil or an undesired migration into groundwater thereof (Wanner 2021).

To date, only a few studies have been conducted that aim to analyse the complex phenomenon of sorption between microplastics and ILs, particularly in the context of investigating the integrity of the ionic pairs (Lisiecka et al. 2023a). It has been suggested that hydrophilic anions are not sorbed on the surface of polypropylene microplastics (Yang et al. 2018). Yet, the transformation of herbicidal ILs containing a surface-active hydrophobic cation and its effects in the presence of microplastics on the environment thereof have not yet been investigated.

Therefore, the following study was conducted to investigate the hypothesis whether ILs containing the herbicidal anion [2,4-D] and different cations with surfactant properties, namely cetyltrimethylammonium [CTA] and didecyldimethylammonium [DDA], exist as an ionic pair or rather separately in aquatic environment. Furthermore, the following aspects were interrogated, namely the sorption of the ions on the polyethylene (PE) microplastic, their toxicity, and the effect on the isomerase of *cis*- to *trans*-unsaturated membrane fatty acids as well-established stress biomarkers in *Pseudomonas* bacteria. Finally, the biodegradability was monitored by applying the OECD 301F standard test. Overall, the results presented are intended to open up a broad discussion on whether an elaborately synthesized IL differs from a cation–anion mixture used in the IL synthesis processes.

## Materials and methods

### Synthesis of ILs containing 2,4-D as the anion

**Fig. 1:**
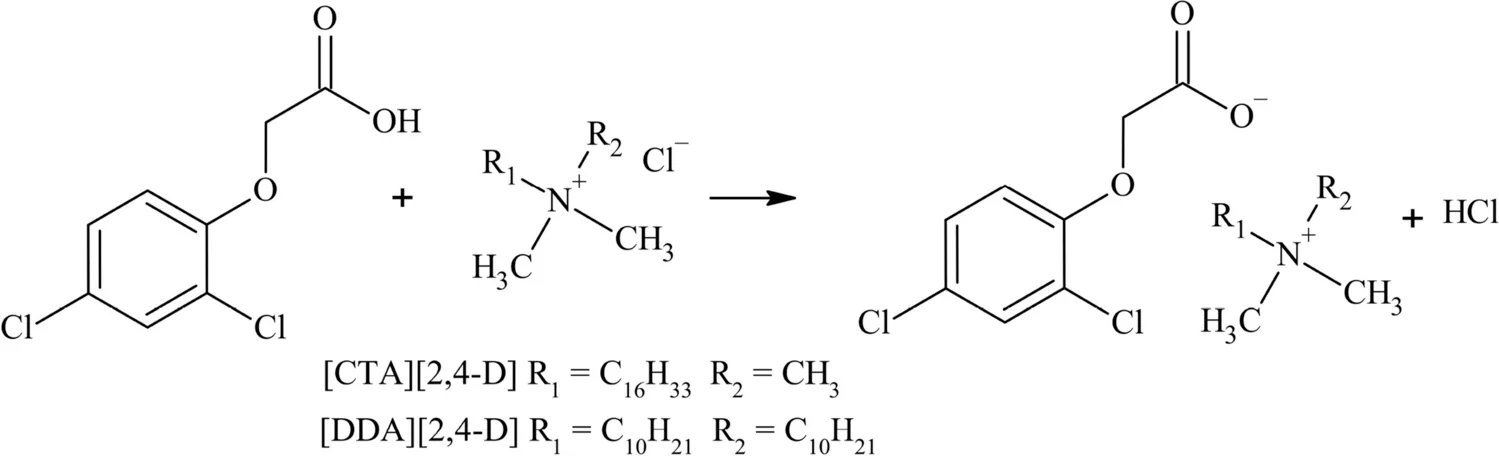
Synthetic pathway of herbicidal ILs with 2,4-D anion

The synthesis of ILs containing the herbicidal anion 2,4-D and the surface-active cations, namely [DDA][2,4-D] (didecyldimethylammonium 2,4-dichlorophenoxyacetate) and [CTA][2,4-D] (cetyltrimethylammonium 2,4-dichlorophenoxyacetate), were described previously in Pernak et al. (2012). The reaction followed the scheme shown in Fig. 1. For the purposes of this study, a freshly crystallized 2,4-dichlorophenoxyacetic acid was used, while the cations were commercially available quaternary ammonium halogens. The synthesis of ILs used within this study is presented in the electronic supplementary information (ESI).

### Determination of the effective half-maximal concentration of ILs and their precursors

The effective half-maximal concentration (EC_50_) was analysed in order to determine the toxic character of [CTA][2,4-D] and [DDA][2,4-D] as well as the following IL precursors: tetraalkylammonium chlorides containing respective hydrophobic cations ([CTA][Cl] and [DDA][Cl]), the sodium salt of the utilized herbicide [Na][2,4-D], as well as the mixture of these chlorides with the herbicidal sodium salt [CTA][Cl] + [Na][2,4-D] and [DDA][Cl] + [Na][2,4-D]. At the beginning of the analyses, 200 µL of a *Pseudomonas putida* KT2440 (DSM 6125) suspension in mineral medium (Niemczak et al. 2017) was placed into a sterile 96-well cell culture plate with OD_560_ = 0.100 ± 0.010. This was incubated in a multi-spectral microchannel analyser Synergy HTX (Biotek, Winooski, VT, USA) at the temperature of 30 °C with continuous shaking until logarithmic growth (OD_560_ approx. 0.350 ± 0.010) was achieved. Subsequently, 50 µL of the aquatic solution of analysed substances was added to each microchannel to obtain the following concentrations: 0.055, 0.027, 0.014, 0.003, 0.001, 0.0003, and 0.00003 mM. In the case of biotic control, the microorganisms were cultivated without the analysed substances, and 50 µL of sterile water was added. For the purposes of abiotic control, a sterile medium with the analysed substances but without microorganisms was prepared. After the bacterial culture reached a stationary phase, on the basis of the OD_560_, the linear regression was obtained for the growth of microorganisms, and their growth rate *G*_R_ was calculated (Eq. (1)):

1 $${G}_{\mathrm{R}}=\frac{\mathrm{ln}\left(ODtx\right)-\mathrm{ln}\left(ODty\right)}{t}$$

where:

*G*_R_: the growth rate of microorganisms,
ODtx: the optical density in *x* time when substances were added,
ODty: the optical density in *y* time after substances were added,
*t*: the growth time.

And inhibition of the microorganism growth *G*_I_ was calculated (Eq. (2)):

2 $${G}_{\mathrm{I}}=\frac{{G}_{\mathrm{R}}\mathrm{sample}}{{G}_{\mathrm{R}}\mathrm{control}}\times 100$$

where:

*G*_I_ inhibition of the growth of microorganisms (%).

The EC_50_/*t* was analysed based on the relationship between the inhibition of the microorganism growth and the concentration of the applied substance according to Syguda et al. (2020).

### Characterization of the PE microplastic

The low-density polyethylene powder (500 µm) was purchased from Thermo Fisher Scientific (Waltham, MA, USA). For identifying the functional groups on the surface of microplastic, a spectroscopic analysis was performed using Fourier-transform infrared spectroscopy (FT-IR) in accordance with Cai et al. (2019). Microplastic samples of PE were prepared in a pastille form (1 mg microplastic + 100 mg KBr), and FT-IR spectra were recorded in the range of 4000–400 cm^−1^ using the Jasco LE 4600 spectrometer (Jasco Inc., Easton, MD, USA).

On the basis of Wang et al. (2021), to determine the porous structure properties (surface area (*A*_BET_), *S*_p_) and total pore volume (*V*_p_), low-temperature N_2_ sorption at − 196 °C was conducted using an ASAP 2020 physisorption analyser (Micromeritics Instrument Co., Norcross, CA, USA). The surface area, pore size distribution, and total pore volume were determined based on the Brunauer–Emmett–Teller (BET) model and utilizing the Barrett-Joyner-Halenda (BJH) method with the Halsey equation (Lisiecka et al. 2024). Before measurement, the analyzed materials underwent degassing at 80 °C for 10 h.

### Sorption of ILs and their precursors on the PE microplastic

The sorption experiment for the selected substances (ILs, their precursors, and cation–anion mixtures thereof) was conducted in accordance with OECD Guidelines for the Testing of Chemicals—Test No. 106 (OECD Guidelines No. 106) and Lisiecka et al. (2023b). To determine the sorption efficiency, specific amounts of PE microplastics, based on Hu et al. (2021), Liu et al. (2019), and Wang et al. (2019), were chosen as follows: 1, 2, 5, 10, 20, 50, 100, and 200 g L^−1^. In each sample, 15 mL of the analysed solutions was pipetted into a glass Erlenmeyer flask. The corresponding amount of microplastic was then added to each solution. All herbicidal solutions were prepared to have the EC_50_ concentration, determined in the section “Determination of the effective half-maximal concentration of ILs and their precursors”. Control experiments were conducted with appropriate substances but without microplastic (containing only the solution, to investigate whether sorption occurred on the glass) and containing only water. Subsequently, the samples were shaken on an orbital shaker at 230 rpm at a temperature of 20 ± 1 °C in the dark to prevent possible photodegradation. After 24 h, the samples were filtered using a syringe filter PTFE 0.22 µm (Chemland, Stargard, Poland). The analysis of the adsorbed cation and anion was carried out using LC–MS/MS (section “LC–MS/MS determination of ILs and their precursors”). Based on the obtained results, the effectiveness of adsorption on microplastics was determined (Parus et al. 2022).

### LC–MS/MS determination of ILs and their precursors

The LC–MS/MS analysis in accordance with Woźniak-Karczewska et al. (2022) was performed on the UltiMate 3000 RSLC chromatographic system from Dionex (Sunnyvale, CA, USA) coupled with the API 4000 QTRAP triple quadrupole mass spectrometer from AB Sciex (Foster City, CA, USA). The determination of ionic liquids was done separately for the [DDA] and [CTA] cations and the [2,4-D] anion using the same analytical column, i.e. a Gemini-NX C_18_ column (100 mm × 2.0 mm I.D.; 3 µm) from Phenomenex (Torrance, CA, USA) thermostated at 35 °C. In both analyses, chromatographic conditions were the same: sample injection volume 5 µL, isocratic elution with 90% methanol containing 5 mM ammonium acetate at a flow rate of 3 mL min^−1^, and run time 4 min. The effluent from the column was directed to the electrospray ionization source (the Turbo Ion Spray) which was operated in a positive ion mode for the cations and in a negative ion mode for the anion. The following settings of the source parameters were applied: curtain gas 10 psi, nebulizer gas 40 psi, auxiliary gas 45 psi, temperature 450 °C, ion spray voltage + / − 4500 V, and collision gas medium. The quantitation limits of the method were 0.04 µg mL^−1^, 0.01 µg mL^−1^, and 0.002 µg mL^−1^ for [DDA], [CTA], and [2,4-D], respectively. Linearity was confirmed in the range of 0.1–2 µg mL^−1^ (*r*^2^ = 0.9998), 0.1–2 µg mL^−1^ (*r*^2^ = 0.9990), and 0.005–0.2 µg mL^−1^ (*r*^2^ = 0.9998) for [DDA], [CTA], and [2,4-D], respectively. Recovery and precision were not tested as sample preparation involved only a dilution step.

### Evaluation of the toxicity of ILs and their precursors after sorption

Following the sorption experiment, the impact of the selected substances on the strain *Pseudomonas putida* KT2440 was assessed through a toxicity analysis, as detailed in Piotrowska et al. (2017). Initially, a bacterial pre-culture was established. After overnight incubation, the bacterial suspension was transferred to a fresh medium with an OD_560_ value of 0.100 ± 0.010. Upon reaching exponential growth (OD_560_ approx. 0.350 ± 0.010), the bacterial culture was centrifuged and washed three times with a phosphate buffer (50 mM, pH 7.0). The resulting pellet was used in the medium containing the supernatants from the sorption experiment. Microorganism growth was monitored for 3 h, measuring turbidity at OD_560_ by analyser Synergy HTX (Biotek, Winooski, VT, USA). To maintain controlled variables in the study, the following cultures were established: microorganisms with the initial solution added (without undergoing the sorption experiment), bacteria without toxicants, the sole mineral medium, and a medium with the analyzed substances without added microorganisms. Regression models representing the growth of the studied microorganisms were derived based on these results.

### Membrane lipid extraction and fatty acid analysis

Fatty acid samples were prepared according to a described procedure (Bligh and Dyer 1959; Morrison and Smith 1964). Briefly, bacterial cells were collected for membrane lipid extraction using chloroform/methanol/water (Bligh and Dyer 1959). Fatty acids were then esterified with methanol by incubation at 80 °C for 15 min in boron trifluoride/methanol (Morrison and Smith 1964). The resulting fatty acid methyl esters (FAME) were extracted in hexane. Subsequently, the analysis of FAME composition was carried out on a gas chromatograph CP-9000 (Chrompack, Middelburg, Netherlands) with a flame ionization detector (GC-FID). Fatty acids were identified with the assistance of provided standards, and the relative amounts of fatty acids were determined based on the peak areas of methyl esters using the Chromatopac integrator C-R6A (Shimadzu, Kyoto, Japan). The *trans/cis* ratio of unsaturated fatty acids was defined as the ratio between the amount of the two *trans*-unsaturated fatty acids (16:1*trans*, 18:1*trans*) and the two *cis* unsaturated fatty acids (16:1*cis*, 18:1*cis*) present in these bacteria’s membrane.

### Assessment of mineralization of ILs and their precursors

The mineralization experiment was conducted in accordance with the OECD 301F guidelines (Pernak et al. 2015; Strotmann et al. 2023). Biological oxygen demand (BOD) was measured every 24 h for 28 days using the OxiTop system (WTW GmbH, Weilheim, Germany) in a thermostated incubator (IKA, Staufen, Germany) covered in an aluminium foil. The activated sludge was collected from the local wastewater treatment plant (Koziegłowy, Poland). Before its application, the activated sludge was aerated for 7 days (pH = 7.2) on a mineral medium which was also utilized later in the experimental stages. The experiment was conducted in bottles made out of brown glass containing the mineral medium and inoculum, with a cell density of approx. 10^6^ cells per mL determined by the plastic Paddle Tester (Hach, Loveland, CO, USA). PE microplastic was added at a concentration of 10 mg L^−1^, according to previous studies (Hu et al. 2021; Lekše et al. 2023; Wang et al. 2020a, b). Additionally, the experiment included the analysis of precursors, herbicidal ILs, and mixtures of precursors separately, each at a concentration of 20–30 mg L^−1^, equivalent to 100 mg L^−1^ of theoretical oxygen demand (ThOD).

Notably, this was calculated based on Eq. (3) for the chemical formula C_c_H_h_N_n_O_o_P_p_. In the case of the IL precursors, the value of ThOD was calculated for the cation without including the inorganic anion.

3 $$ThOD=\frac{16\left[2c+\frac{1}{2}\left(h-3n\right)+\frac{5}{2}p-o\right]}{\text{molecular mass of the test substance}}$$

In order to halt the nitrification, allylthiourea (1.16 mg L^−1^) was added into the bottles. The gas-impermeable bottles were equipped with a CO_2_ trap (solid NaOH) and incubated in darkness at 20 °C for 28 days. The efficiency of mineralization was calculated based on the sampling of carbon dioxide from each of the bottles (measured automatically through the electronic head OxiTop). All the samples were analysed alongside the controls (mineral medium without inoculum, studied substances in medium without inoculum) as well as the blank samples (medium and inoculum without studied substances). On the basis of the results, the primary degradation was calculated using Eq. (4) based on OECD 301 F guideline:

4 $${D}_{\mathrm{t}}= \frac{{S}_{\mathrm{b}}-{S}_{\mathrm{a}}}{{S}_{b}}\times 100$$

where:

*D*_t_: primary degradation at time *t* (28 days) (%),
*S*_a_: the amount of residue of the test chemical in the inoculated medium at the end of the test (mg).
*S*_b_: the amount of residue of the test chemical in the abiotic control at the end of the test (mg).

### Statistical analysis

The statistical analysis was completed using the single-variable variation analysis (ANOVA) (where *p* < 0.05). The presented error bars represent the obtained standard error for the mean (*n* = 3).

## Results

### Synthesis of ILs containing 2,4-D as the anion

ILs were obtained in the metathesis reaction with 91–99% yield. The synthesized products were soluble in DMSO, alcohols, and chloroform. However, their solubility in water was notably limited due to the substantial hydrophobicity of the utilized cations (Pernak et al. 2012). The structures of the analyzed substances are presented in Table 1.

**Table 1:** Structures of ILs with 2,4-D anion.

Acronym	Systematic name and structural pattern	Yield [%]	Appearance at 25 °C	Reference
[CTA][2,4-D]	cetyltrimethylammonium 2,4-dichlorophenoxyacetate	99	grease	(Pernak et al. 2012)
[DDA][2,4-D]	didecyldimethylammonium 2,4-dichlorophenoxyacetate	91	liquid	(Pernak et al. 2012)

### Determination of the effective half-maximal concentration (EC_50_)

The results of the performed studies aimed to assess the EC_50_ concentrations, causing a 50% decrease in the growth of the bacteria, for the analysed compounds (Table 2). It was observed that the herbicidal anion 2,4-D did not exhibit any toxic characteristics toward the model bacterial strain *Pseudomonas putida* KT2440 within the analysed concentration range, which was in agreement with previous observations (Fanous et al. 2007; Somasundaram et al. 1990 and Woźniak-Karczewska et al. 2022).

**Table 2:** Determined EC_50_ values for *Pseudomonas putida* KT2440

Compound	Classification	EC_50_ (µmol L^−1^)
[CTA][2,4-D]	IL	23.28 ± 3.28
[CTA][Cl]	Precursor	21.60 ± 2.34
[CTA][Cl] + [Na][2,4-D]	Mixture of precursors	23.52 ± 1.41
[DDA][2,4-D]	IL	13.49 ± 1.70
[DDA][Cl]	Precursor	10.59 ± 1.01
[DDA][Cl] + [Na][2,4-D]	Mixture of precursors	12.36 ± 2.14
[Na][2,4-D]	Precursor	Non-toxic in the tested concentration range

Furthermore, the EC_50_ values obtained for the ILs were coincidentally similar to those of the corresponding chlorides and the cation–anion mixtures, respectively. This means that the surface-active cation has an influence on the toxicity of the entire substance, no matter if it was introduced as a salt with a chloride anion or as previously synthesized ILs.

### Characterization of the PE microplastic

**Fig. 2:**
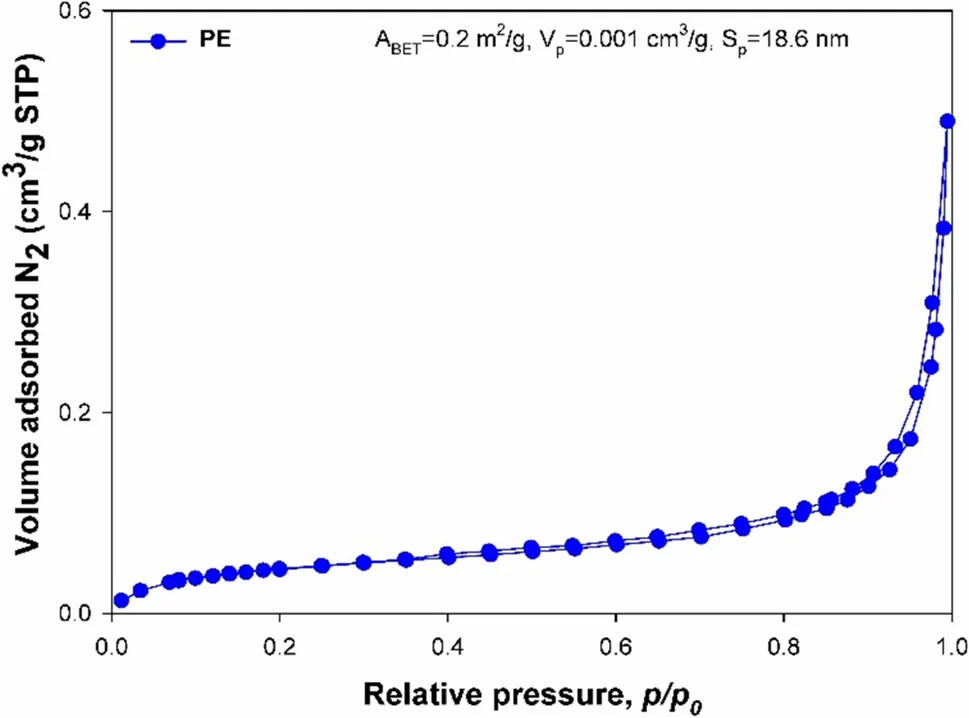
N_2_ adsorption/desorption isotherms of polyethylene microplastic

The PE microplastic used in the study was characterized using FT-IR spectroscopy and BET isotherms. The FT-IR spectra were conducted to confirm the presence of functional groups in the PE microplastic. As shown in Figure S1, the peak wavenumbers associated with the methyl group (2929 cm^−1^) and the methylene radical (2843 and 1470 cm^−1^) establish the identity of the analyzed microplastics as polyethylene (PE). The textural results indicated that the N_2_ sorption isotherms of the PE microplastic samples (Fig. 2) can be classified as type II with a type H4 hysteresis loop, typical for macroporous structures with a low value of the BET surface area of 0.2 m^2^ g^−1^. The average pore diameter (Sp) was 0.001 cm^3^ g^−1^, and the total pore volume (Vp) of PE was equal to 18.6 nm.

### Sorption of ILs and their precursors on PE microplastic

The sorption experiment evidently shows that the selected cations, [CTA] and [DDA], which display surface-active properties, were indeed sorbed on the surface of the PE microplastic (Table 3).

**Table 3:** Adsorption (%) of cations of ILs and precursors on the surface of PE microplastic

Compound	Amount of PE microplastic (g L^−1^)							
	1	2	5	10	20	50	100	200
[CTA][2,4-D]	6.6 ± 1.0	10.6 ± 0.9	15.8 ± 0.6	19.7 ± 1.0	32.7 ± 2.5	41.0 ± 1.4	51.9 ± 1.7	57.1 ± 1.4
[CTA][Cl]	5.9 ± 1.5	8.1 ± 0.7	13.3 ± 1.5	21.5 ± 1.6	27.0 ± 1.7	44.7 ± 2.0	53.8 ± 1.3	59.5 ± 2.5
[CTA][Cl] + [Na][2,4-D]	5.1 ± 1.0	10.9 ± 0.9	15.7 ± 1.0	18.2 ± 1.1	27.9 ± 1.8	37.0 ± 1.5	49.3 ± 1.3	54.6 ± 0.7
[DDA][2,4-D]	4.4 ± 1.2	7.6 ± 1.3	10.2 ± 1.2	13.4 ± 1.0	20.2 ± 1.2	37.1 ± 1.6	44.8 ± 1.7	46.8 ± 1.5
[DDA][Cl]	3.3 ± 0.9	7.5 ± 0.8	9.0 ± 0.9	12.8 ± 1.0	17.1 ± 1.0	29.7 ± 1.7	41.9 ± 2.2	48.7 ± 1.4
[DDA][Cl] + [Na][2,4-D]	1.7 ± 0.5	6.9 ± 1.2	9.6 ± 1.7	13.5 ± 1.8	20.7 ± 1.2	36.8 ± 0.8	44.4 ± 1.3	47.9 ± 1.2
[Na][2,4-D]	-	-	-	-	-	-	-	-

(-) not detected; the results of adsorption (%) of anion 2,4-D of compounds on the surface of PE microplastic were included in ESI (Table S1)

In the case of surface-active cations (Table 3), it was noted that as the amount of PE microplastic sorbent increases, the amount of sorbed cation also increases. In the chosen range of microplastic for the experiment (from 1 to 200 g L^−1^), the sorption values obtained extended between 5 and 60% for [CTA] cation and between 2 and 48% for [DDA] cation.

### Evaluation of the toxicity of ILs and their precursors after sorption

**Fig. 3:**
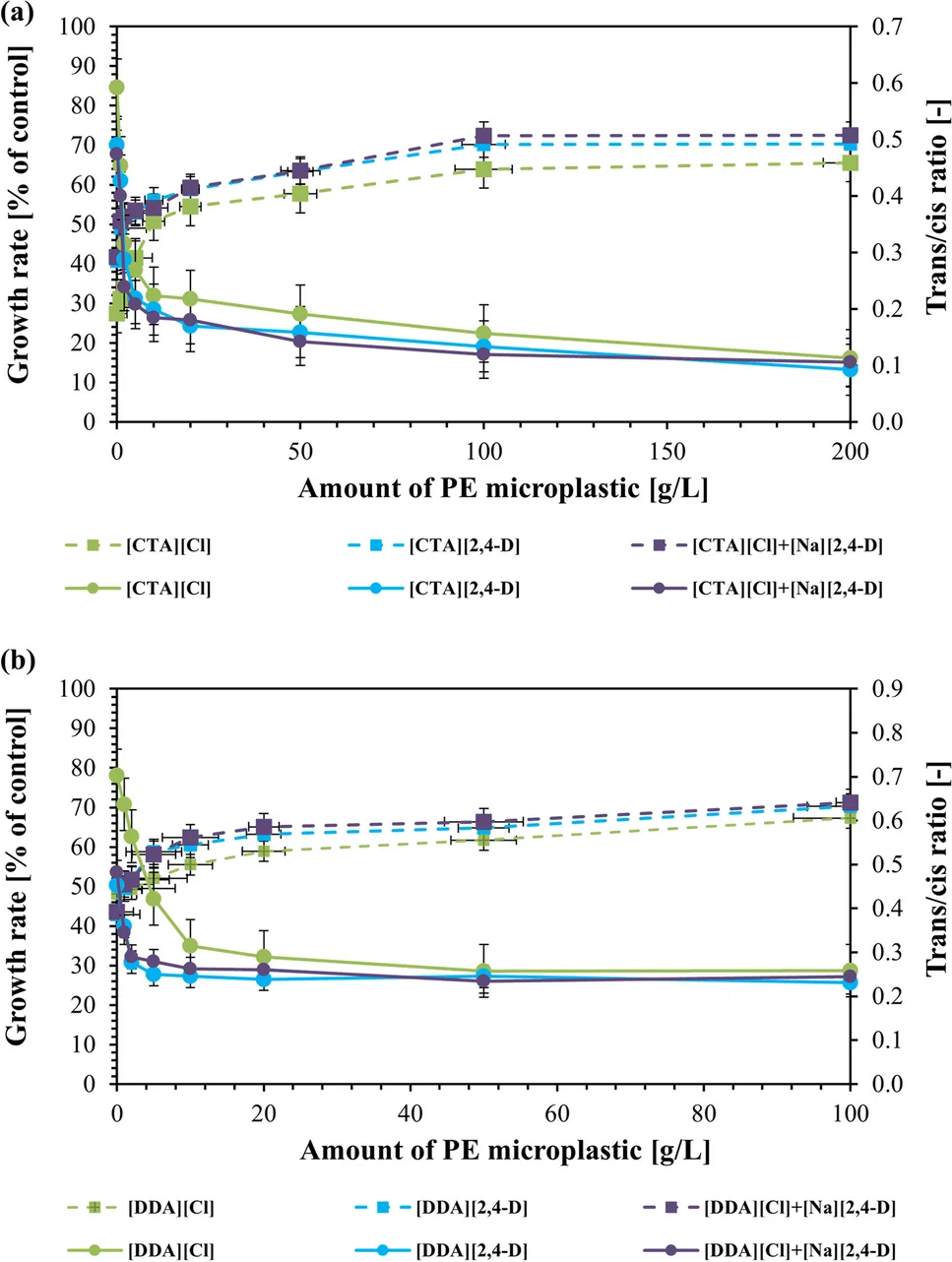
Effect of ILs and their precursors with [CTA] cation **a** and [DDA] cation **b** on growth (  ) and *trans/cis* ratio of unsaturated fatty acids (  ) of *Pseudomonas putida* KT2440 in the presence of PE microplastic. All compounds used were in EC_50_ concentrations determined within this study

In order to further consider the fate of the ILs after the sorption process occurred, the outcomes of the toxicity studies for *Pseudomonas putida* KT2440 were presented. Hence, the experimental results (Fig. 3) suggest that there is indeed a toxic effect of the surface-active cation, which inhibited growth of the bacteria. This can be attributed to interactions on the cellular level which affect the integrity and increase the permeability of the cytoplasmic membranes. As a result of this, an undesirable ion exchange occurs and the cells are destroyed (Chapman et al. 1993; Glover et al. 1999; Zhou et al. 2016).

### Membrane lipid extraction and fatty acid analysis

The presence of surface-active cations in the analysed samples, due to its toxic influence on the bacterial strain of *Pseudomonas putida* KT2440, caused the change in the configuration of the fatty acids (Fig. 3). Figure 3 clearly shows that there is a direct relation between the amount of PE microplastic and the *trans/cis* ratios. An increased amount of PE microplastic caused a decrease in cationic toxicity and limited the changes in the isomerization of unsaturated fatty acids. The ratio range varied between 0.1 and 0.7, which was characterized by similar values for ILs, chlorides, and precursor mixtures. This once again highlights the lack of anionic influence on the toxicity of the cation in the ILs. Notably, this undermines the ability to modify the ionic pair properties.

### Assessment of mineralization of ILs and their precursors

**Fig. 4:**
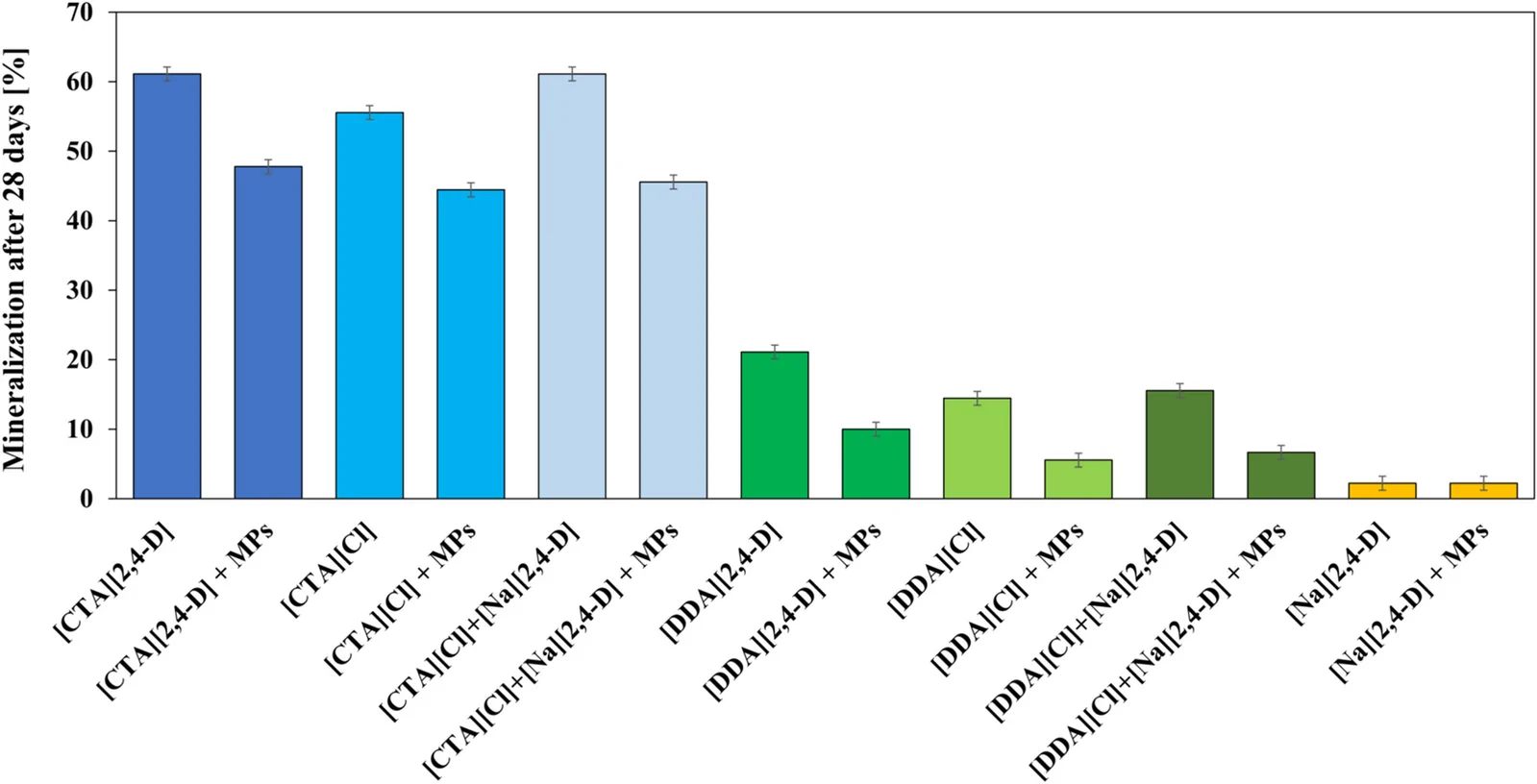
Mineralization of investigated substances in the presence of PE microplastic (in a concentration of 10 mg L.^−1^)

The activated sludge, which was utilized in this study, had the ability of surfactant mineralization. This was confirmed in this study, as well as described by others who emphasized the degradation potential of sediments in a wastewater treatment plant due to their continuous contact with surfactant pollutants from fabric softeners, hair care products, or detergents (Wang et al. 2018). Therefore, the evaluation of the primary biodegradation, monitored for 28 days, was performed on the respective ILs, their precursors, as well as their cation–anion mixtures thereof. In Fig. 4, it can be observed that a partial mineralization of surface-active cations took place. A higher mineralization efficiency was noted for substances containing the [CTA] cation in comparison to those containing the [DDA] cation. Moreover, from the obtained results, one can conclude the negligible ability of degradation of the 2,4-D anion by the activated sludge.

## Discussion

The studies conducted have demonstrated that the presence of the non-toxic herbicidal anion does not reduce the toxicity of the surface-active cation. This finding contradicts the theoretical assumption that the combined effects of the cation and anion would enhance the properties of the synthesized ionic pair (Pernak et al. 2012; Niemczak et al. 2017; Syguda et al. 2020). Regardless of the tested setups, the toxicity of the analyzed ionic liquids (ILs) depends solely on the properties of the cation when paired with a non-toxic anion. The potential interactions between the cation and anion in the resulting ion pairs did not affect the interactions of these cations with the bacterial cell membrane—the cell lysis mechanism remained unaffected. This indicates that both ions behave as dissociated and solvation-separated chemical entities. This observation aligns with current theories on ILs, suggesting that modeling parameters such as toxicity in ionic liquids through a random combination of cations and anions, as commonly found in aqueous solutions, is not feasible. These ions exhibit behaviour as independent chemical entities, as described by numerous previous studies (Lisiecka et al. 2023a, 2023b; Parus et al. 2022; Wilms et al. 2023b, 2023a; Woźniak-Karczewska et al. 2022).

The adsorption of cations on the microplastic resulted from the hydrophobic interactions, which are considered the dominant mechanism allowing sorption of hydrophobic organic substances on the microplastic surface (Llorca et al. 2018; Wu et al. 2019; Tourinho et al. 2019) as well as electrostatic interactions which are created by the attraction or repulsion of the particles with opposite charges (Wang et al. 2015; Xu et al. 2018; Guo et al. 2018; Razanajatovo et al. 2018; Tourinho et al. 2019). Furthermore, the results for the ILs, the chlorides, and the cation–anion mixtures suggest that there are no serious differences between the studied substances. Notably, the sorption of the cation occurred similarly in each of the setups which corresponds to Lisiecka et al. (2023a). Moreover, the presence of the surface-active cation has not caused an increase of the anion sorption on the microplastic surface, which did not exceed 5% for the highest concentrations of microplastics (Table S1), as also demonstrated in Fatema and Farenhorst (2022).

It is important to highlight that the sorption of surface-active substances on microplastic surfaces poses a potential environmental threat. This is due to the increased risk of prolonged impact of pollutants in aquatic environment (Llorca et al. 2018; Lisiecka et al. 2023a). Microplastics which are laden with adsorbed hydrophobic pollutants could act as potential carriers of various toxic substances, like pesticides, pharmaceuticals, perfluoroalkyl substances (PFAS), and surfactants (Ateia et al. 2020; Xia et al. 2020). As microplastics are released into water, the xenobiotics previously adsorbed to their surfaces can migrate and adversely affect aquatic organisms, potentially harming entire ecosystems (Tourinho et al. 2019). Thus, understanding the consequences of the sorption of cationic surfactants used in ILs and as adjuvants in commercial herbicides on microplastics is crucial for monitoring and managing this potential threat in aquatic and terrestrial environments (Xia et al. 2020; Shen et al. 2021; Jiang et al. 2022). The obtained sorption results clearly show the lack of interactions between the cation and the anion in the herbicidal ILs. This is supported by recent literature on the absence of ionic integrity in ILs based on herbicidal anions such as 2,4-D, dicamba, and glyphosate (Woźniak-Karczewska et al. 2022; Parus et al. 2022; Wilms et al. 2023a, 2023b; Lisiecka et al. 2023b). This raises doubts about the necessity of the costly and complex synthesis of ILs if the results are analogous to those of the precursor mixtures.

The adsorption of surface-active cations to polyethylene (PE) microplastics due to hydrophobic interactions led to a decrease in their concentrations in the supernatant. Reduced bioavailability of the cations resulted in a dose-dependent decrease in bacterial strain inhibition by the PE microplastics (Fig. 3). This supports the well-established notion that increasing the concentration of ILs and surface-active cations limits the growth of microorganisms (Piotrowska et al. 2017; Parus et al. 2020; Lisiecka et al. 2023b; Homa et al. 2024). The phenomenon of sorption on microplastics reduced the toxic effect on bacterial growth and also influenced the results obtained in trans/cis isomerization, a cellular response to stress conditions. The changes in the fatty acid composition are a protection mechanism present in several Gram-negative bacteria (Heipieper et al. 1992, 2007; Cronan 2002; Zhang and Rock 2008; Molina-Santiago et al. 2017; Eberlein et al. 2018). The conversion of the unsaturated *cis*-fatty acids to the corresponding *trans* configuration occurs due to stresses acting on the bacterial cell (Heipieper et al. 1992, 1996; Duldhardt et al. 2010; Piotrowska et al. 2016, 2017; Kotchaplai et al. 2017; Eberlein et al. 2018) and is a regularly used molecular marker for membrane stress in *Pseudomonas* species.

Additionally, the presence of PE microplastics in the analyzed systems reduced the mineralization efficacy of the cations, likely due to sorption phenomena on the microplastic surface, as described in the section “Determination of the effective half-maximal concentration of ILs and their precursors”. Sorption of Herbicidal ILs on PE Microplastic, as well as biofilm formation on the microplastic surface, limits the diffusion of xenobiotics into the biofilm (Kostka and Nealson 1995; Flemming and Wingender 2010; Dang and Lovell 2016; Flemming and Wuertz 2019). Moreover, in the biodegradation studies, from the obtained results, one can conclude the negligible ability of degradation of the 2,4-D anion by the activated sludge. This shows that the 2,4-D anion is not an attractive source of carbon for the selected microorganisms in the analysed time, as suggested by Orhon et al. (1989), however, in contrast to observations of other studies (Bolana 1996; Estrella et al. 1993; Ławniczak et al. 2015; Zipper et al. 1999) about the rapid degradation of the 2,4-D anion.

The degradation of 2,4-D by microbial communities can indeed vary significantly depending on the environment and microbial composition. In terms of bacterial diversity capable of degrading 2,4-D, studies have identified several genera involved in the process, including *Sphingomonas*, *Pseudomonas*, *Cupriavidus*, and *Achromobacter*. These bacteria possess specific degradation pathways, often initiated by the *tfdA* gene, which encodes an enzyme responsible for breaking down 2,4-D (Gonod et al. 2006; Chen et al. 2024). However, the presence and abundance of these bacteria in activated sludge can be highly variable.

The limited degradation observed in our study could be due to the absence or low prevalence of these specific bacterial strains in the sludge samples we analyzed. This suggests that the degradation potential for 2,4-D might indeed be underrepresented in typical activated sludge microbiomes, especially in systems not previously exposed to the herbicide. Several reports indicate that the degradation of 2,4-D is more effective in environments with a history of herbicide application, where microbial communities have had the opportunity to evolve the necessary catabolic pathways (Kat et al. 1994; Marrón-Montiel et al. 2006; Zabaloy et al. 2010). Moreover, this validates the findings from Wilms et al. (2020a), which indicate that the herbicide anion MCPA was not mineralized by the activated sludge, contrasting with the hydrophobic cation.

The obtained results for ILs, their precursors, as well as the cation–anion mixtures suggest that only cations are capable of influencing the mineralization of the analysed substances. Therefore, it is evident that the biodegradation of the cations occurred independently to the attached anion. Moreover, the combination of the herbicidal anion with the hydrophobic cation did not allow an increased biodegradative potential of ILs. Hence, there is no doubt that the limited contemporary studies confirming the biodegradability of ILs have failed to analyse the significant integrity of the ion pair.

This study shows that ILs containing a surface-active cation and herbicidal anion in aqueous environment behave as chemically separate entities. Notably, there were no observed changes in sorption, toxicity, and mineralization between individual ions and ions constituting the analysed ILs. The only dissimilarity relates to the differing sorption of cations, depending on their hydrophobicity and toxicity, respectively. The herbicide anion, on the other hand, was not sorbed, regardless of the type of cation. The ions interacted with PE microplastic, *Pseudomonas putida* KT244D and activated sludge independently of each other, and the combination into ILs did not change the properties of the substance.

The obtained results show the lack of benefit of synthesizing and purifying herbicidal ionic liquids. Hence, this study can be used as an introductory argument to debating the ecological and economic issues regarding the production of herbicidal ILs. However, it is still necessary to verify whether other ILs, that can potentially enter the environment, are capable of forming an ionic pair with improved agrichemical properties. A highly important aspect of our research is the evaluation of the adsorption of cations characterized by surface-active properties on PE microplastic, which evidently affected the behaviour of the analysed ions. The adsorption of the cations directly reduced the toxic effect against the bacteria and altered the fatty acid profiles thereof. Moreover, due to the use of activated sludge, the evident decrease in the mineralization of hydrophobic cations was confirmed.

Based on the findings of this study, it is recommended to deepen investigations by exploring other types of microplastics beyond polyethylene (PE), which was the sole focus in this research. Different microplastics may exhibit distinct chemical properties that could influence their interactions with ionic liquids (ILs). Furthermore, future research should encompass a wider range of ILs with varied molecular structures, as it has been confirmed that these may interact differently with microplastics. Additionally, expanding the study to include other environmental variables—such as the presence of organic matter (e.g. humic acids) and other contaminants—could provide insights into their significant influence on the sorption process of ILs onto microplastics (Ateia et al. 2020).

In this experimental setup, a range of microplastic concentrations from 1 to 200 g/L was selected, which aligns with the commonly used values in the literature (Liu et al. 2019; Wang et al. 2019; Hu et al. 2021). Typically, sorption studies utilize a single concentration, such as 1 g/L (Jiang et al. 2020) or 100 g/L (Ni et al. 2023). Although some research has explored concentrations below 1 g/L (Wang et al. 2020a, 2022; Liu et al. 2022), this study employed multiple concentrations to assess how varying levels of microplastics affect the sorption behavior of the substances under aqueous conditions. This approach allowed us to capture a broader understanding of concentration-dependent interactions under selected experimental conditions.

PE microplastics display the ability to sorb xenobiotics, influencing and limiting their bioavailability while also extending the persistence of pollutants in the environment. The accumulation of toxic xenobiotics on the microplastic’s surface in both aqueous and soil environments is an undesirable phenomenon. According to the current knowledge, there is no unambiguous information regarding desorption and release of previously adsorbed xenobiotics. Therefore, microplastics, which act as additional adsorbents in the environment, are becoming a new element of the ecosystem, which must be taken into careful consideration in the context of the interactions between pollutants and microorganisms.

## Supplementary Information

Below is the link to the electronic supplementary material.

Supplementary file1 (PDF 173 KB)

## Data Availability

The authors state that data supporting the results of this study are available in the article, supplementary information files, and RepOD Data: 10.18150/GZTSPO.
