# Supplementary material for: Effect of microplastic on sorption, toxicity, and mineralization of 2,4-dichlorophenoxyacetic acid ionic liquids
Source: Appl Microbiol Biotechnol. 2024 Nov 26;108:523. doi: 10.1007/s00253-024-13353-6 (PMC11599340; doi:10.1007/s00253-024-13353-6)
Supplement: Supplementary file 1 [file 253_2024_13353_MOESM1_ESM.pdf]

Supporting Information for:

Effect of microplastic on sorption, toxicity and mineralization of 2,4-dichlorophenoxyacetic acid Ionic Liquids

Natalia Lisiecka[1,2], Marta Woźniak-Karczewska[1*], Anna Parus[1], Maria Simpson[1], Robert Frankowski[3], Agnieszka Zgoła-Grześkowiak[3], Katarzyna Siwińska-Ciesielczyk[1], Michał Niemczak[1], Christian Eberlein[2], Hermann J. Heipieper[2], Łukasz Chrzanowski[1,2]

[1] Institute of Chemical Technology and Engineering, Poznan University of Technology, Berdychowo 4, 60-965 Poznan, Poland
[2] Department of Molecular Environmental Biotechnology, Helmholtz Centre for Environmental Research – UFZ, Permoserstraße 15, 04318 Leipzig, Germany
[3] Institute of Chemistry and Technical Electrochemistry, Poznan University of Technology, Berdychowo 4, 60-965 Poznan, Poland

Corresponding author: marta.wozniak-karczewska@put.poznan.pl

Key words: polyethylene, herbicides, 2,4-dichlorophenoxyacetic acid, *Pseudomonas putida* KT2440, antimicrobial activity

Synthesis of herbicidal ILs containing 2,4-D as the anion

Cetyltrimethylammonium chloride (50% aqueous solution) was purchased from Stockmeier Chemia (Poznań, Poland), didecyldimethylammonium chloride (Arquad 2.10−50, 50% aqueous solution) was purchased from Akzo Nobel (Amsterdam, Netherlands). Before use, quaternary ammonium salts were isolated form aqueous solutions via evaporation of the solvent in a rotary evaporator followed by drying in a vacuum dryer at 50 ºC for 24 h.

Firstly, (2,4-dichlorophenoxy)acetic acid (0.01 M), distilled water (20 mL) and a 10% aqueous solution of NaOH (0.011 M) were mixed in a round-bottom flask equipped with a magnetic stirrer, a reflux condenser and an additional funnel. The mixture was heated at 50 °C until a clear solution was obtained. Quaternary ammonium chloride or bromide (0.01 M), which was previously dissolved in water (30 mL), was then added and stirred for 30 min at room temperature. The product was then extracted from the aqueous phase with chloroform (50 mL) and washed with distilled water until chloride or bromide ions were no longer detected with $AgNO_3$. After removal of the chloroform, the product was dried under reduced pressure at 70 °C for 24 h (Pernak et al., 2012).

Characterization of the PE microplastic

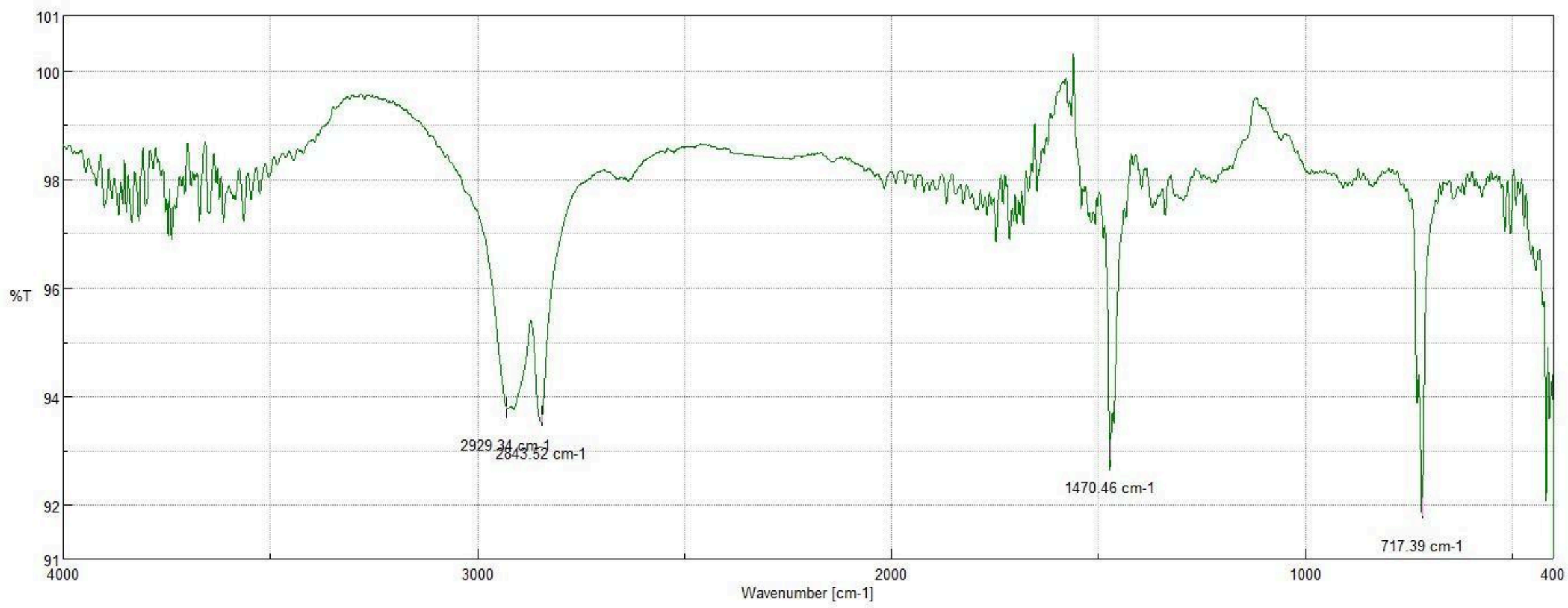

Figure S1. FT-IR spectra analysis of PE microplastic

Sorption of herbicidal ILs on microplastic

Table S1. Adsorption [%] of anion 2,4-D of compounds on the surface of PE microplastic.

**Compound**	**Amount of PE microplastic [g L^-1]**							
	**1**	**2**	**5**	**10**	**20**	**50**	**100**	**200**
[CTA]**[2,4-D]**	0.0 ± 0.0	0.0 ± 0.0	0.0 ± 0.0	0.0 ± 0.0	0.3 ± 0.6	0.8 ± 0.9	4.9 ± 2.2	5.9 ± 2.0
[CTA][Cl]	-	-	-	-	-	-	-	-
[CTA][Cl]+[Na]**[2,4-D]**	0.0 ± 0.0	0.0 ± 0.0	0.0 ± 0.0	0.0 ± 0.0	0.0 ± 0.0	0.0 ± 0.0	4.3 ± 0.9	5.7 ± 0.5
[DDA]**[2,4-D]**	0.0 ± 0.0	0.0 ± 0.0	0.0 ± 0.0	0.0 ± 0.0	0.0 ± 0.0	0.0 ± 0.0	3.3 ± 1.9	4.4 ± 0.8
[DDA][Cl]	-	-	-	-	-	-	-	-
[DDA][Cl]+[Na]**[2,4-D]**	0.0 ± 0.0	0.0 ± 0.0	0.0 ± 0.0	0.0 ± 0.0	0.0 ± 0.0	0.0 ± 0.0	2.5 ± 2.2	5.7 ± 0.8
[Na]**[2,4-D]**	0.0 ± 0.0	0.0 ± 0.0	0.0 ± 0.0	0.0 ± 0.0	0.0 ± 0.0	0.0 ± 0.0	0.0 ± 0.0	0.0 ± 0.0
